# Measurement properties of tools measuring mental health knowledge: a systematic review

**DOI:** 10.1186/s12888-016-1012-5

**Published:** 2016-08-23

**Authors:** Yifeng Wei, Patrick J. McGrath, Jill Hayden, Stan Kutcher

**Affiliations:** 1 Sun Life Financial Chair in Adolescent Mental Health team, IWK Health Centre, 5850 University Ave., P.O Box 9700, Halifax, NS B3K 6R8 Canada; 2 IWK Health Centre, Nova Scotia Health Authority and Dalhousie University, 5850 University Ave., P.O Box 9700, Halifax, NS B3K 6R8 Canada; 3 Centre for Clinical Research, Room 403, 5790 University Avenue, Halifax, NS B3H IV7 Canada

**Keywords:** Mental health literacy, Measurement tools, Psychometrics, Systematic review, Mental health knowledge

## Abstract

**Background:**

Mental health literacy has received great attention recently to improve mental health knowledge, decrease stigma and enhance help-seeking behaviors. We conducted a systematic review to critically appraise the qualities of studies evaluating the measurement properties of mental health knowledge tools and the quality of included measurement properties.

**Methods:**

We searched PubMed, PsycINFO, EMBASE, CINAHL, the Cochrane Library, and ERIC for studies addressing psychometrics of mental health knowledge tools and published in English. We applied the COSMIN checklist to assess the methodological quality of each study as “excellent”, “good”, “fair”, or “indeterminate”. We ranked the level of evidence of the overall quality of each measurement property across studies as “strong”, “moderate”, “limited”, “conflicting”, or “unknown”.

**Results:**

We identified 16 mental health knowledge tools in 17 studies, addressing reliability, validity, responsiveness or measurement errors. The methodological quality of included studies ranged from “poor” to “excellent” including 6 studies addressing the content validity, internal consistency or structural validity demonstrating “excellent” quality. We found strong evidence of the content validity or internal consistency of 6 tools; moderate evidence of the internal consistency, the content validity or the reliability of 8 tools; and limited evidence of the reliability, the structural validity, the criterion validity, or the construct validity of 12 tools.

**Conclusions:**

Both the methodological qualities of included studies and the overall evidence of measurement properties are mixed. Based on the current evidence, we recommend that researchers consider using tools with measurement properties of strong or moderate evidence that also reached the threshold for positive ratings according to COSMIN checklist.

## Background

Mental disorders affect approximately 1 in 5 people [1, 2]. They are the leading cause of the global burden of diseases with the highest proportion of burden occurring in people aged 10–29 years [3]. Without appropriate treatment, they result in significant negative impacts on both short and long term social, economic and interpersonal outcomes as well as increasing risk for all causes of early age mortality, including suicide [4]. A recent international cross-sectional study in 17 countries further demonstrated that mental disorders are associated with increased risks of the onset of a wide range of chronic physical conditions (e.g., heart disease, stroke, cancer, diabetes mellitus, hypertension, asthma, other chronic lung diseases, and peptic ulcer) [5]. Effective treatments are available, but are uncommonly accessed by most youth with mental disorders [6, 7]. A recent systematic review found that barriers to receipt of mental health care include lack of knowledge about mental illness and stigma related to mental illness [8].

Mental health literacy has been considered as an effective approach to address these identified challenges and it is foundational for mental health promotion, early identification and treatment of mental disorders [9–11]. Mental health literacy includes 4 components: 1) knowledge about how to obtain and maintain good mental health; 2) knowledge about mental disorders and their treatments; 3) decreasing stigma against those living with mental disorders; and 4) enhancing help-seeking efficacy [12]. Research shows that improved mental health literacy may be able to promote early identification of mental disorders, improve mental health outcomes, increase the use of health services, and enable the community to take actions to achieve better mental health [13–16].

Mental health literacy is a derivative of health literacy that evolved from functional literacy applied in health care environments addressing treatment adherence to a broader framework that further includes social and cognitive skills to improve and maintain good health and it is considered as an empowerment tool in social and political contexts [17]. According to World Health Organization (WHO)[18], health literacy is a significant independent determinant of health, it is: “a stronger predictor of an individual’s health status than income, employment status, education and racial or ethnic group.” (page 7).

Numerous mental health literacy programs have been developed over the last two decades. For example, a recent systematic review identified 27 studies evaluating the effectiveness of mental health literacy programs in the secondary school setting, in which 15 specifically addressed mental health knowledge about mental disorders, and the rest of studies focused on stigma and help-seeking behaviors [19]. Another systematic review of reviews analyzed approximately 500 school mental health interventions most of which addressed the promotion of positive mental health [20]. Further, a meta-analysis of a particular mental health literacy intervention, mental health first aid, has shown its impact on knowledge about mental disorders and help-seeking resources [21]. However, there is a paucity of evaluations of the tools to measure mental health literacy. For example, many mental health knowledge evaluation tools used in mental health literacy studies are varied in content, purpose, and quality, which may lead to non-comparable study results and increase risk of biased conclusions. Although sometimes the content of a mental health knowledge tool may be specifically designed to be somewhat different from another depending on the local community in which it is deployed, tools used must be of acceptable quality as the use of tools with poor quality may result in non-evidenced and unreliable results when evaluating the effectiveness of mental health literacy interventions or investigating mental health literacy levels in order to develop appropriate interventions in the community.

We previously conducted a scoping review to summarize and categorize currently available mental health literacy tools, however, we did not synthesize information on the psychometric properties of the included tools or assess the quality of the evidence available [22]. This report bridges that gap by critically appraising the quality of studies evaluating the measurement properties of tools addressing knowledge about mental disorders, the quality of included measurement properties and determining the level of evidence of overall quality of measurement properties of applied tools. Such a review will help researchers to identify what/how measurement properties of a mental health knowledge tool can be validated in a psychometric study. It will further help the research community to better choose appropriate tools to evaluate existing mental health literacy interventions or guide the development of new interventions. We will report the quality of mental health literacy tools addressing stigma and help-seeking in separate reviews.

## Methods

We followed the protocol recommended by the Preferred Reporting Items for Systematic Reviews and Meta-Analyses (PRISMA) (http://www.prisma-statement.org/) to report findings. We adapted and applied the Consensus-based Standards for the selection of health Measurement Instruments (COSMIN) checklist manual for the critical appraisal of studies [23] and quality criteria for embedded measurement properties developed by the same group of professionals [24]. COSMIN checklist is a robust tool developed specifically for systematic reviews on psychometric studies.

### Search strategy

We searched the following bibliographic databases: PubMed, PsycINFO, EMBASE, CINAHL, the Cochrane Library, and ERIC, using four sets of search terms from the scoping review [22], with the consultation of a health librarian between January and June 2015, and further updated and extended the search in Feb and March of 2016 to identify relevant studies. Appendix 1 is an example of the search strategies applied in PubMed. In addition, to ensure as much as possible that we would not miss relevant publications, we also searched Google Scholar, using the names of included knowledge tools identified from the search and finally, we also checked reference lists of included studies for additional studies. Two authors of this review were experts in mental health literacy field and they contributed to ensure that relevant studies were included.

Two people from the research team applied an iterative process to independently screen titles (stage 1); titles of remaining studies to further exclude irrelevant studies, abstracts or brief scanning of full texts if abstract reviewing was not sufficient to make decisions of inclusion (stage 2); and full texts of citations identified in the electronic literature search (stage 3). Reference check and Google Scholar search were conducted following these 3 stages of search. Following this, they met to compare their final included articles, and review and decide together the inclusion of articles one reviewer didn’t include but the other reviewer did. A systematic review methodologist and two mental health professionals (also authors of this review) were available to guide the search, data analysis and help making final decisions on included studies.

### Selection criteria

We included any quantitative studies that evaluated measurement properties (reliability, validity or responsiveness) of mental health knowledge tools. Studies for inclusion had to report not only the psychometrics of the tool but also the statistical analysis used to evaluate the tool. We focused on tools that address mental health in general or common mental disorders that typically onset during adolescent years, including depression, anxiety, Attention Deficit Hyperactivity Disorder (ADHD) and schizophrenia. Our search did not restrict the publication dates or the age of participants.

We excluded studies addressing substance use disorder although it is common among youth due to the fact that it covers a wide range of sub areas, and it requires an independent research strategy beyond the scope of our current study. We excluded studies that were not in English and those that only reported the psychometrics of tools but did not describe the statistical analysis used to evaluate the tools. For examples, many studies only reported the Chronbach’s alpha but did not describe how this was achieved and therefore there were no data available for the quality assessment.

### Data extraction

We used the COSMIN checklist manual [23] to develop a data extraction form. According to the COSMIN checklist [23], a systematic review of studies on measurement properties could cover any of the following 9 areas in 3 dimensions. This includes: 1. Reliability (e.g., internal consistency, reliability (e.g., test-retest, intra-rater reliability, and measurement error); 2. Validity (content validity, structural validity (e.g., factor analysis), hypothesis testing (construct validity), cross-cultural validity, and criterion validity); and 3. Responsiveness (e.g., sensitivity to change). In addition, we followed the COSMIN checklist recommendation to document the population (e.g., age and gender), setting (e.g., country and culture), tool content and format, as well as types of psychometrics assessed in the included studies.

### Study quality assessment (risk of bias assessment)

We applied the COSMIN checklist with a 4-point scale [23] to assess the methodological quality of each available study for each measurement property. The COSMIN checklist has 7–18 items to assess the study design and statistical methods for each property, with each item ranked as “excellent”, “good”, “fair”, or “poor” (see COSMIN checklist: http://www.cosmin.nl/). The overall methodological quality of each study assessing a measurement property is ranked as “excellent”, “good”, “fair, or “poor” by taking the lowest rating of any item in a box (worst score counts). For example, the domain for a study assessing the internal consistency contains 11 items for evaluation. If any one of the 11 items is scored “poor” but the rest of the 10 items are scored “excellent”, “good”, or “fair”, the final score for the study on internal consistency is “poor”.

### Levels of evidence of overall quality

The level of evidence of the overall study quality of a measurement property was determined by the methodological quality of the available studies as determined by the COSMIN checklist stated above [23] and the consistency of the quality of measurement properties (positive (+), negative (-), indeterminate (?) findings) [24]. The details of the criteria for the quality of each measurement property can be found in Appendix 2. These criteria for the level of overall evidence were informed by Terwee and colleagues [23, 24] as refined in a systematic review of questionnaires measuring continuity of care and Cochrane Back & Neck Group’s recommendations on the overall quality of the evidence of each assessed outcome [25, 26] (Appendix 3). As a result, the overall quality rating of a measurement property across studies were then determined with 5 levels of evidence: strong (+++ or ---), moderate (++ or --), limited (+ or -), conflicting (+/-) or unknown (x) (Appendix 3). The unknown (x) rating includes studies of poor methodological quality, as well as studies in which the quality of measurement properties were rated as “indeterminate” regardless of the study quality.

In March and April of 2016, two reviewers separately rated the quality of studies, the quality of each measurement property, and synthesized the levels of overall quality of measurement properties. Both reviewers studied and discussed the ranking system to make sure they were confident about its application. They compared and discussed their final rankings of the included studies and measurement properties. An Excel data ranking form was created for each level of analysis to store and keep track of quality scores for each reviewer. For rankings confirmation when they did not agree, a systematic review methodologist and two mental health professionals (also authors of this review) were available to solve the differences between the two reviewers.

Based on the overall level of evidence, we consider measurement properties with strong positive ratings (+++) as ideal; moderate positive ratings (++) as preferred; and limited positive ratings (+) as minimally acceptable for use in research and practice. However, tools with measurement properties of negative ratings (---, --, -), or conflicting ratings (+/-), or unknown (x) have yet to be further studied before application since the quality of these properties was under the threshold or indeterminate defined by Terwee and colleagues regardless of the study quality [24].

## Results

**Fig. 1:**
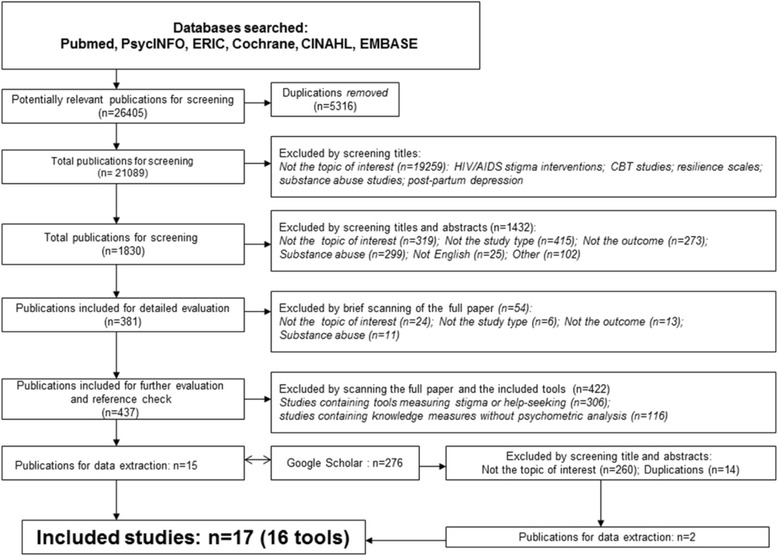
Flow chart of search results

Figure 1 demonstrates the flow chart of search results. As described in Methods section, we first checked study titles and screened out duplicates and studies unrelated to our topic of interest, such as studies measuring HIV/AIDS interventions, cognitive behavioural therapies, resilience programs, or knowledge about other specific mental disorders (e.g., post-partum depression, eating disorders, autism) which were not the focus of our current review. The data were then imported into Reference 2.0 database management software and more duplicates were removed [27]. We further checked both titles and abstracts and screened out studies based on criteria in the first stage, as well as non-English publications. This procedure was repeated until the last stage of full text scanning and we excluded studies addressing other aspects of mental health literacy: stigma and help-seeking. As a result, we identified 131 studies that contained tools measuring mental health knowledge in which 17 studies provided psychometrics analysis of 16 tools applied in these studies. Our analysis focused on the psychometrics of these 16 knowledge measurement tools, which are: Knowledge about Schizophrenia Questionnaire, Knowledge about Schizophrenia Test, Multiple-Choice Knowledge of Mental Illnesses Test, Mental Health Knowledge Schedule, Depression Multiple Choice Question, Depression Literacy, Anxiety Literacy, Test of Knowledge About ADHD, Knowledge about Depression and Mania Inventory, Journey of Hope Outcome Survey, Knowledge of Mental Disorders, Adolescent Depression Knowledge Questionnaire, Mental Health Disorder Recognition questionnaire, Mental Health Knowledge Questionnaire, Knowledge Questionnaire on Home Care of Schizophrenics, and Mental Health Literacy Scale [28–44]. This includes 2 studies [35, 36] assessing Depression Literacy; another 2 studies assessing Knowledge about Schizophrenia Test [30, 32] and one study [35] evaluating 2 tools (Depression Literacy & Anxiety Literacy) in this current review.

### Study characteristics

We described the detailed study characteristics in Table 1. The 16 tools evaluated mental health knowledge among different populations: community members [30, 33, 43, 44]; mental health patients [28, 34, 38]; patients’ family members and caregivers [29, 30, 32, 38, 40]; police officers [30, 31]; mental health professionals [30, 32, 34]; high school students [41–44]; post-secondary students [39]; athletes [35]; immigrants [36]; or elementary teachers [37]. The tools addressed either mental health knowledge in general [31, 33, 39–41, 43, 44], or knowledge about specific mental disorders, such as depression [34–36, 38, 42], schizophrenia [28–30, 32], anxiety [35], and ADHD [37].

**Table 1:** Study characteristics

Author/year	Measurement tool	Description of tool	Population of study	Age of study participants	Study sample size	Country of study	Mental health knowledge type	Psychometric properties of tool assessed
1. Ascher-Svanum & Krause, 1999 [28]	Knowledge about Schizophrenia Questionnaire (KASQ)	25 multiple-choice questions on knowledge of mental illness and management	Inpatients	M = 35 (SD = 11.4); (range: 18–58)	N = 53 (study 1); N = 53 (study 2); N = 10 (study 3); N = 20 (study 4)	US	Schizophrenia	Internal consistency; Reliability; Responsiveness (Sensitivity to change); Content validity
2. Balasubramanian et al., 2013 [29]	Knowledge Questionnaire on Home Care of Schizophrenics (KQHS)	32 item multiple choice questionnaire on four aspects of home care	Home care givers	Unknown	N = 21	India	Schizophrenia	Content validity; Internal consistency
3. Compton et al., 2007 [30]	Knowledge about Schizophrenia Test (KAST)	21 multiple choice questions on knowledge of schizophrenia	Community members; Families of people with schizophrenia; police officers; mental health professionals	M = 43.7 (SD = 12.1) (Community members); M = 44.0 (SD = 12.8) (families); M = 37.8 (SD = 7.8) (police officers); M = 44.2 (SD = 10.1) (mental health professional)	N = 144 (community members); N = 77 (families members); N = 170 (police officers); N = 50 (mental health professionals)	US	Schizophrenia	Internal consistency; Construct validity (hypothesis testing); Content validity; Criterion/concurrent validity
4. Compton et al., 2011 [31]	Multiple-Choice Knowledge of Mental Illnesses Test (MC-KOMIT)	33 multiple-choice items on knowledge of common mental illnesses	Police officers	M = 38.3 (SD = 8.4)	199	US	General knowledge	Internal consistency; Reliability; Construct validity (hypothesis testing); Content validity; Responsiveness
5. Daltio et al., 2015 [32]	Knowledge about Schizophrenia Test (KAST)	17 multiple choice questions on knowledge of schizophrenia	Caregivers of patients with schizophrenia, and patients of other conditions; mental health clinicians	M = 56.05 (SD = 12.9) (caregivers)	N = 89 caregivers of patients with schizophrenia; N = 30 caregivers of general patients; N = 30 mental health professionals	Portugal	Schizophrenia	Content validity; Cross-cultural validity; Reliability Construct validity
6. Evans-Lacko et al., 2010 [33]	Mental Health Knowledge Schedule (MAKS)	6-point Likert scale on 12 items of stigma knowledge of mental illness	General public	25–45	N = 92 (study 1); N = 37 (study 2); N = 403 (study 3)	UK	General knowledge	Internal consistency; reliability; Content validity
7. Gabriel & Violato, 2009 [34]	Depression Multiple Choice Question (MCQ)	27 multiple-choice items on knowledge of depression	Patients and psychiatrists	M = 43 (SD = 11.3) (range: 18–65) (patients); M = 52 (SD = 11.6) (Psychiatrists)	N = 63 (patients)	Canada	Depression	Internal consistency; Content validity; Convergent validity; Structural validity (factor analysis)
					N = 12 (psychiatrists)			
8. Gulliver et al., 2012 [35]	Depression Literacy (D-Lit)	22 true/false items on knowledge of depression	Elite athletes	M = 25.5 (median = 24.5) (range: 18–48)	N = 40 (study 1); N = 12 (study 2)	Australia	Depression	Internal consistency; Reliability
	Anxiety Literacy Questionnaire (A-Lit)						Anxiety	
9. Kiropoulos et al., 2011 [36]	Depression Literacy (D-Lit)	22 true/false items on knowledge about depression	Immigrants	M = 65.4 (SD = 8.57) (range: 48–88)	202	Australia	Depression	Internal consistency; Reliability
10. Hepperlen et al., 2002 [37]	Test of Knowledge About ADHD (KADD)	22 error-choice items to assess knowledge and attitudes toward students with ADHD	Elementary school teachers	M = 39.43 (SD = 9.05)	103	US	ADHD	Internal consistency; Content validity; Structural validity (factor analysis);
11. Kronmuller et al., 2008 [38]	Knowledge about Depression and Mania Inventory (KDMI)	44 true/false items on knowledge of Depression and Mania	Patients and relatives	M = 45.2 (SD = 13.6) (range: 18–82); M = 47.4 (SD = 14.5) (range: 19–80)	N = 112 (patients); N = 89 (relatives)	Germany	Depression	Concurrent/criterion validity; Hypothesis testing (Discriminative validity); Content validity; Responsiveness
12. O’Connor & Casey, 2015 [39]	Mental Health Literacy Scale (MHLS)	Multiple choice on 35 items regarding knowledge and attitudes about help-seeking, and ability to recognize disorders	First year university students (S)	M = 21.10 ± 6.27 (S); M = 33.09 ± 8.01	372 (S); 43 (M)	Australia	General knowledge	Internal consistency; Reliability; Measurement error; Content validity; Structural validity; Construct validity
			Mental health professionals (M)					
13. Pickett-Schenk et al., 2000 [40]	Journey of Hope (JOH) Outcome Survey	4-point Likert scale on 15 items on mental health knowledge	Family members of people with mental illness	M = 56.48	424	US	General knowledge	Internal consistency; Construct validity (hypothesis testing); Structural validity (Factor analysis);
14. Serra et al., 2013 [41]	Knowledge of Mental Disorders (KMD)	“Yes, “No”, and “I don’t know” responses to assess knowledge on the name and characteristics of mental disorders and ability to distinguish them from somatic illnesses	High school students	M = 17.3 (SD = 1.3); (range: 15–24)	1,023	Italy	General knowledge	Internal consistency; Structural validity (factor analysis); Construct validity (hypothesis testing)
15. Hart et al., 2014 [42]	Adolescent Depression Knowledge Questionnaire (ADKQ)	13 dichotomous and 2 fill-in-the- blank questions on depression knowledge	Grade 9 students	Not reported	8,216	US	Depression	Internal consistency; Structural validity (factor analysis)
16. Swami et al., 2011 [43]	Mental health disorder recognition questionnaire (MDRQ)	7-point Likert scale on 20 statements of mental illness descriptions in which 15 are real and 5 are foils	General public	M = 38.11 (SD = 14.89)	477	UK	General knowledge	Reliability; Construct validity (hypothesis testing); Convergent validity
17. Wang et al., 2013 [44]	Mental Health Knowledge Questionnaire (MHKQ)	“yes”, and “no” responses to 20 general mental health knowledge questions	Community members	M = 50 (SD = 17)	1953	China	General knowledge	Internal consistency; Factor analysis

*M* mean, *SD* standard deviation

Fourteen tools focused on facts about mental illness, such as the etiology, diagnoses, prevalence, signs/symptoms, and comorbidity; as well as knowledge about treatments/side effects and mental health services [28–35, 37–40, 42, 44]. Of these 14 tools, 1 (Mental Health Knowledge Schedule) further included stigma-related knowledge on help-seeking, recognition, support, and employment [33]; 1 (Knowledge about Depression and Mania Inventory) addressed knowledge about coping and illness management [38], and 1 (Knowledge about Schizophrenia Questionnaire) included knowledge about legal issues pertaining to mental illness [28]. Two tools (Knowledge of Mental Disorders, Mental health disorder recognition questionnaire) measured participants’ ability to identify the illness appropriately [41, 43].

Table 1 indicates that 15 out of 17 included studies were conducted in Western countries with 35 % of the studies conducted in the United States of (*n* = 6), followed by Australia (*n* = 3), United Kingdom (*n* = 2), Canada (*n* = 1), Germany (*n* = 1), Italy (*n* = 1), and Portugal (*n* = 1). Two studies took place in non Western countries, China (*n* = 1) and India (*n* = 1). Study participants varied across studies and some studies included various types of participants, such as: family members of care givers of people with mental illness (*n* = 5), community members (*n* = 4), patients of mental illness (*n* = 3), mental health professionals (*n* = 3), police (*n* = 2), high school students (*n* = 2), university students (*n* = 1), elementary school teachers (*n* = 1), immigrants (*n* = 1), and athletes (*n* = 1).

### Methodological quality of studies

Table 2 presents the methodological quality per study on each measurement property of a measurement tool. The 16 tools assessed properties such as internal consistency (15 tools) [28–31, 33–35, 37–44], content validity (10 tools) [28–31, 33, 34, 37–39, 42], construct validity (hypothesis testing) (7 tools) [30, 31, 34, 38, 40, 41, 43], reliability (8 tools) [28, 30, 31, 33, 35, 39, 42, 43], structural validity/factor analysis (6 tools) [34, 37, 40–42, 44], criterion validity (2 tools) [30, 38], responsiveness (sensitivity to change) (3 tools) [28, 31, 38] and cultural validity (1 tool) [32]. The methodological quality of included studies ranged mostly from “poor” to “good” (*n* = 11) except that 5 studies addressing content validity [30, 31, 33, 38, 39], and 1 study [39] addressing internal consistency and structural validity demonstrated “excellent” quality. More than half (*n* = 9) of the studies evaluating internal consistency were ranked as having “poor” quality while the rest were rated as “good” [34, 37, 40–42, 44]. Studies evaluating reliability (*n* = 8) also had mixed qualities ranging from “poor” to “good”. Studies evaluating structural (*n* = 6) and construct (hypothesis testing) (*n* = 7) validity mostly demonstrated “fair” quality. All studies (*n* = 3) examining responsiveness (sensitivity to change) were scored as having “poor” quality. One study was identified as assessing cultural validity with “fair” quality [32]. One study was identified assessing measurement errors with “good” quality [39].

**Table 2:** Methodological quality of a study on each measurement property of a measurement tool

Measurement tool	Study Author	Internal consistency	Reliability	Content validity	Measurement errors	Structural validity	Criterion validity	Cultural validity	Hypothesis testing	Responsiveness
1. Knowledge about Schizophrenia Questionnaire (KASQ) [28]	Ascher-Svanum & Krause, 1999	Poor	Poor	Poor						Poor
2. Knowledge Questionnaire on Home Care of Schizophrenics (KQHS) [29]	Balasubramanian et al., 2013	Poor		Good						
3. Knowledge about Schizophrenia Test (KAST) [30, 32]	Compton et al., 2007	Poor		Excellent			Fair		Fair	
	Daltio et al., 2015		Fair	Excellent				Fair	Fair	
4. Multiple-Choice Knowledge of Mental Illnesses Test (MC-KOMIT) [31]	Compton et al., 2011	Poor	Good	Excellent					Fair	Poor
5. Mental Health Knowledge Schedule (MAKS) [33]	Evans-Lacko et al., 2010	Poor	Fair	Excellent						
6. Depression Multiple Choice Question (DMCQ) [34]	Gabriel & Violato, 2009	Good		Poor		Fair			Fair	
7. Depression Literacy (D-Lit) [35, 36]	Gulliver et al., 2012	Poor	Poor							
	Kiropoulos et al., 2011	Poor	Fair							
8. Anxiety Literacy Questionnaire (A-Lit) [35]	Gulliver et al., 2012	Poor	Poor							
9. Test of Knowledge About ADHD (KADD) [37]	Hepperlen et al., 2002	Good		Poor		Fair				
10. Knowledge about Depression and Mania Inventory (KDMI) [38]	Kronmuller et al., 2008	Poor		Excel-lent			Fair		Fair	Poor
11. Mental Health Literacy Scale (MHLS) [39]	O’Connor & Casey, 2015	Excel-lent	Good	Excel-lent	Good	Excel-lent			Fair	
12. Journey of Hope (JOH) Outcome Survey [40]	Pickett-Schenk et al., 2000	Good				Fair			Fair	
13. Knowledge of Mental Disorders (KMD) [41]	Serra et al., 2013	Good				Fair			Fair	
14. Adolescent Depression Knowledge Questionnaire (ADKQ) [42]	Hart et al., 2014	Good		Poor		Fair				
15. Mental health disorder recognition questionnaire (MDRQ) [43]	Swami et al., 2011		Fair						Fair	
16. Mental Health Knowledge Questionnaire (MHKQ) [44]	Wang et al., 2013	Good							Fair	

Based on the quality criteria determined from use of the COSMIN checklist [23], study quality was downgraded if there were deficiencies of study design. For example, we found most (*n* = 16) [28–38; 40–44] studies didn’t report the percentage of missing items or described how missing items were handled, which may have introduced bias in their results [45], and therefore downgraded the study quality. Additionally, more than half of the studies (*n* = 11) [28–33, 35, 36, 38, 43, 44] evaluated the internal consistency without checking unidimensionality of the tool resulting in “poor” quality of the study on this measurement property. The 2 studies [30, 38] evaluating criterion validity were rated as “fair” also due to the lack of justification regarding the “gold standard” the tool was compared against. Further, all studies evaluating construct validity (hypothesis testing) (*n* = 10) [30–32, 34, 38–41, 43, 44] were rated as “fair” mostly because studies did not formulate the hypothesis “a priori”, or the hypothesis was vague without specifying what was expected. And lastly, the “poor” quality of responsiveness (*n* = 3) (sensitivity to change) [28, 31, 38] was mostly attributable to the application of inappropriate statistics such as effect sizes or *t-test* statistics.

### Quality of measurement properties

While Table 2 presents the study quality, Table 3 presents the quality of each measurement property of all 16 tools. In terms of measurement properties by each tool (results by cases in the table), they all demonstrated mixed quality (+, -, or ?) as Table 3 demonstrated. When we investigated the quality by the measurement property (results by columns in the table), responsiveness received positive ratings (+) (above the quality criteria threshold) in all 3 studies it was evaluated [28, 31, 38]. The construct validity received positive ratings in all 8 studies it was evaluated [30, 31, 34, 38–41, 43], except that of 1 tool [43] with indeterminate (?) rating. The criterion validity evaluated in 2 studies [30, 38] demonstrated negative ratings (-) (below the quality criteria threshold). The rest of the measurement properties all demonstrated mixed ratings (+, -, or ?).

**Table 3:** Quality of each measurement property

Measurement tool	Study Author	Internal consistency	Reliability	Content validity	Measurement error	Structural validity	Criterion validity	Cultural validity	Hypothesis testing	Responsiveness
1. Knowledge about Schizophrenia Questionnaire (KASQ) [28]	Ascher-Svanum & Krause, 1999	?	+	?						+
2. Knowledge Questionnaire on Home Care of Schizophrenics (KQHS) [29]	Balasubramanian et al., 2013	?		+						
3. Knowledge about Schizophrenia Test (KAST) [30, 32]	Compton et al., 2007	?		+			-		+	
	Daltio, et al., 2015		-	+				N/A	+	
4. Multiple-Choice Knowledge of Mental Illnesses Test (MC-KOMIT) [31]	Compton et al., 2011	?	-	+					+	+
5. Mental Health Knowledge Schedule (MAKS) [33]	Evans-Lacko et al., 2010	-	+							
6. Depression Multiple Choice Question (DMCQ) [34]	Gabriel & Violato, 2009	-		?		+			+	
7. Depression Literacy (D-Lit) [35, 36]	Gulliver et al., 2012	?	-							
	Kiropoulos et al., 2011	?	+							
8. Anxiety Literacy Questionnaire (A-Lit) [35]	Gulliver et al., 2012	?	+							
9. Test of Knowledge About ADHD (KADD) [37]	Hepperlen et al., 2002	+		?		-				
10. Knowledge about Depression and Mania Inventory (KDMI) [38]	Kronmuller et al., 2008	?		+			-		+	+
11. Mental Health Literacy Scale (MHLS) [39]	O’Connor & Casey, 2015	+	+	+	?	?			+	
12. Journey of Hope (JOH) Outcome Survey [40]	Pickett-Schenk et al., 2000	+				?			+	
13. Knowledge of Mental Disorders (KMD) [41]	Serra et al., 2013	-				?			+	
14. Adolescent Depression Knowledge Questionnaire (ADKQ) [42]	Hart et al., 2014	+		?		?				
15. Mental health disorder recognition questionnaire (MDRQ) [43]	Swami et al., 2011		+						?	
16. Mental Health Knowledge Questionnaire (MHKQ) [44]	Wang et al., 2013	-							+	

+: positive rating, -: negative rating, ?: indeterminate rating, N/A: no information provided

### Level of evidence of overall quality of measurement properties

Table 4 demonstrates levels of evidence for the overall quality of each measurement property, which was determined by both the methodological quality of each study from Table 2 and the quality of each measurement property from Table 3. The criteria for the levels of evidence were developed to evaluate a measurement property of a tool in different studies. However, our review identified only 2 tools assessed in different studies [30, 32, 35, 36], and the measurement properties for the rest of the 14 tools were assessed in only one study each. Therefore, the overall quality of these tools was based on 1 study only for each tool. Accordingly, two tools [43, 44] demonstrated consistent positive ratings (+ or ++) (limited or moderate evidence) for their measurement properties. Two tools [28, 35] demonstrated unknown (“x”) ratings for all measurement properties (studies of poor methodological quality or indeterminate quality of measurement properties). The rest of the tools showed mixed ratings (x, -, +, +/-, ++, --, +++, ---) of their measurement properties [29–42].

**Table 4:** Overall level of evidence of measurement properties

Measurement tool	Study Author	Internal consistency	Reliability	Content validity	Measurement errors	Structural validity	Criterion validity	Cultural validity	Hypothesis testing	Responsiveness
1. Knowledge about Schizophrenia Questionnaire (KASQ) [28]	Ascher-Svanum & Krause, 1999	x	x	x						x
2. Knowledge Questionnaire on Home Care of Schizophrenics (KQHS) [29]	Balasubramanian et al., 2013	x		++						
3. Knowledge about Schizophrenia Test (KAST) [30, 32]	Compton et al., 2007	x	-	+++			-		+	
	Daltio et al., 2015									
4. Multiple-Choice Knowledge of Mental Illnesses Test (MC-KOMIT) [31]	Compton et al., 2011	x	--	+++					+	x
5. Mental Health Knowledge Schedule (MAKS) [33]	Evans-Lacko et al., 2010	x	+	+++						
6. Depression Multiple Choice Question (MCQ) [34]	Gabriel & Violato, 2009	--		x		+			+	
7. Depression Literacy (D-Lit) [35, 36]	Gulliver et al., 2012	x	+/-							
	Kiropoulos et al., 2011									
8. Anxiety Literacy Questionnaire (A-Lit) [35]	Gulliver et al., 2012	x	x							
9. Test of Knowledge About ADHD (KADD) [37]	Hepperlen et al., 2002	++		x		-				
10. Knowledge about Depression and Mania Inventory (KDMI) [38]	Kronmuller et al., 2008	x		+++			-		+	x
11. Mental Health Literacy Scale (MHLS) [39]	O’Connor & Casey, 2015	+++	++	+++	x	x			+	
12. Journey of Hope (JOH) Outcome Survey [40]	Pickett-Schenk et al., 2000	++				x			+	
13. Knowledge of Mental Disorders (KMD) [41]	Serra et al., 2013	--				x			+	
14. Adolescent Depression Knowledge Questionnaire (ADKQ) [42]	Hart et al., 2014	++		x		x				
15. Mental health disorder recognition questionnaire (MDRQ) [43]	Swami et al., 2011		+						+	
16. Mental Health Knowledge Questionnaire (MHKQ) [44]	Wang et al.,2013	++							+	

*Note*: +++ or --- = strong evidence, ++ or-- = moderate, + or- = limited evidence, +/-: conflict findings; x = studies of poor methodologic quality or studies with indeterminate property quality

In terms of overall ratings by measurement property (results by columns in the table), we found strong evidence (+++) of the content validity of 5 tools [30–33, 38, 39], and of the internal consistency of 1 tool [39]; moderate evidence (++ or --) of the internal consistency of 6 tools [34, 37, 40–42, 44], of the content validity of 1 tool [29], and of the reliability of 2 tools [28, 39]; limited evidence (+ or -) of the reliability of 3 tools [30, 33, 43], the structural validity of 2 tools [41, 42], the criterion validity of 2 tools [30, 38], and the construct validity of 9 tools [30, 31, 34, 38–41, 43, 44]. We also found the level of evidence of a number of measurement properties was unknown (x), including the responsiveness of 3 tools [28, 31, 38]; the internal consistency of 8 tools [28–31, 33, 35, 38]; the reliability of 3 tools [28, 35]; the structural validity of 4 tools [39–42]; the content validity of 4 tools [28, 34, 37, 42], and the measurement error of 1 tool [39].

According to the criteria in Appendix 3, the level of evidence of overall quality for a number of measurement properties was unknown “x” mainly because of poor study quality presented in Table 3, including the failure to assess the dimensionality of the tool which is the prerequisite for a clear interpretation of the internal consistency [46] and relatively small sample sizes (<30). Further, the level of evidence with negative ratings (- or --) was attributed to a number of factors, including the relatively weak correlations of two tools, the Knowledge about Schizophrenia Test and the Knowledge about Depression and Mania Inventory [30, 38] with gold standard tools (<0.70) when assessing the criterion validity; the lower-than-quality-threshold internal consistency (α < 0.7) of Knowledge of Mental Disorders [41], or the failure of one study [37] on the tool Test of Knowledge About ADHD to discuss explained variance when assessing its structural validity.

Based on the level of evidence and criteria described above in the methods section, we recommend the application of 13 measures for their specific properties: Knowledge about Schizophrenia Test, Multiple-Choice Knowledge of Mental Illnesses Test, and Knowledge about Depression and Mania Inventory with their content (+++, Ideal) and construct (+, Acceptable) validity; Mental Health Literacy Scale with its internal consistency and content validity (+++, Ideal), reliability (++, Preferred), and construct validity (+, Acceptable); Mental Health Knowledge Schedule with its content validity (+++, Ideal) and reliability (+, Acceptable); Depression Multiple Choice Question with its structural (+, Acceptable) and construct (+, Acceptable) validity; Test of Knowledge About ADHD with its internal consistency (+, Acceptable); Journey of Hope with its internal consistency (Preferred) and construct (+, Acceptable) validity; Knowledge of Mental Disorders with its construct (+, Acceptable) validity; Adolescent Depression Knowledge Questionnaire with its internal consistency (++, Preferred); Mental Health Disorder Recognition questionnaire with its reliability (+, Acceptable) and construct (+, Acceptable) validity; Mental Health Knowledge Questionnaire with its internal consistency (++, Preferred) and construct (+, Acceptable) validity; and Knowledge Questionnaire on Home Care of Schizophrenics for its content (++, Preferred) validity.

## Discussion

This systematic review evaluated 16 mental health knowledge tools in 17 studies. It has provided a comprehensive critical analysis of the study characteristics, the methodological quality, the quality of individual measurement properties, and the overall evidence of the measurement properties of the included tools.

A review of the study characteristics indicates that most of the studies were conducted among the adult population and there were only four studies targeting youth [33, 35, 36, 38]. This highlights the need for the development, evaluation and validation of tools addressing mental health knowledge specifically for youth who are at a vulnerable period of time related to the risk for developing mental illness. Further, most (*n* = 15) studies were conducted in Western countries and cultural validity of the tools was assessed in only one study. Therefore, at this time it is not possible to determine if measures created in one culture or setting can be appropriately used in another, especially in non-developed countries and regions where culture, social and economic contexts are dramatically different.

A strongly validated tool may not only help to accurately measure the impact of current mental health literacy interventions, but also can guide the development of new interventions. Rising from the assessment of study quality is the question of what constitutes a good psychometric study. Based on our findings and the COSMIN criteria, we propose that such a study may report on a sample size ≥30, examine the internal consistency and the dimensionality of the tool, determine the factors of the tool using factor analysis and explain the variances attributed to the factors, and establish the construct validity by testing pre-designed hypothesis. If it is a new tool, it is important to make sure tool items reflect the construct measured, are relevant to its population and fulfill its purposes. Also, such a study may examine the stability of the tool over appropriate period of time (usually 3 to 6 weeks). When a tool is applied in a culturally different setting, researchers may translate and back translate the tool, consider the adaption of the tool and pilot it in the target population (*n* ≥ 10) before its application.

We recommended mental health knowledge tools by measurement properties because the level of evidence of each property within a tool was different even in the same study, and different tools measured different properties. Therefore, we decided it is not appropriate to conclude that one tool is better than the other. For example, the Mental Health Knowledge Questionnaire [44] was evaluated on two properties (internal consistency and construct validity) and both reached the Acceptable and Preferred level of evidence. Another tool, the Mental Health Literacy Scale [39] was evaluated on six properties, four of which reached Acceptable or above level of evidence and two demonstrated level of evidence Unknown. In this case, we encourage readers to focus on the level of evidence of each individual property as well as their actual needs in practice when choosing which tool to use. Meanwhile, based on what we suggested above, researchers may further need to reach a consensus on what properties should be included for a psychometric study so that readers can compare the quality of different tools and make informed decisions.

However, as the validation of measurement properties is an ongoing and iterative process and needs to be conducted in different settings and contexts with different populations [47]. Further research could find that many of the measurement tools that demonstrated relatively low level of evidence of quality in the current review may have excellent psychometric properties with some populations in future research. More well-designed studies are needed to gather the evidence of the measurement properties to demonstrate their consistency and stability across studies.

The conceptual framework of mental health literacy includes 3 outcomes (knowledge, stigma and help-seeking), of which knowledge about positive mental health is a component. However, our review focused on tools addressing mental illness. We made this decision based on a number of factors. First, positive mental health covers a wide range of topics related to health promotion at individual, family, community and society level [48]. This includes social and emotional learning, resiliency, coping, social and psychological welling, physical health, healthy eating, family relationship and connectedness, school and workplace environment, community involvement, and social support, to name a few. Each topic contains an independent and substantial body of research and unless we specifically come to a consensus on the scope and definition of each sub topic, it is unlikely that we are able to aggregate measurement tools in this area for use in assessments. Also, the mental health literacy concept is relatively new and the filter of each searched database is not sensitive to catch the search terms designed under the mental health literacy framework. We may have to design separate search strategies and conduct separate reviews to address this topic.

Lastly, as noted in the methods section, the COSMIN checklist applied the ‘worse score counts’ approach to determine the methodological quality of a property. This means a poorly scored item weighs more than all other well scored items in a criteria box. This may lead to a less positive score. For example, items in the criteria box for the content validity of DMCQ [34] were all rated as “excellent” on important factors such as constructs to be measured, purpose of the tool, and comprehensiveness of the tools, except one item rated as “poor” due to the failure to assess the relevancy of the tool for the study population. In this case, the final score of “poor” may not adequately reflect the true quality of the study.

### Limitations

We applied the COSMIN checklist originally developed to assess the quality of health status questionnaires and it may not be ideal for mental health knowledge tools in spite of some modifications that we made to the checklist. We didn’t include studies published in other languages, and therefore we may have missed some eligible studies. We only checked Google Scholar for grey literature because other available databases for grey literature such as GreyMatters is designed to contain information for health-related literature (e.g., health economics, clinical trials, drug and device information) and we decided they are not relevant to our topic of interest. However, this decision may have led to missing studies.

## Conclusions

To our knowledge, this review is the first to assess the quality of mental health knowledge measurement tools. We applied a standardized method, the COSMIN checklist, to evaluate quality of studies assessing measurement properties; we further assessed the quality of each measurement property, and provided a comprehensive and critical synthesis of current evidence in the field. The available evidence indicates that both the methodological qualities of included studies and the overall evidence of measurement properties are mixed. Based on the current evidence, we recommend that researchers consider using those knowledge assessment tools with measurement properties of positive ratings with strong and moderate evidence (++, or +++) or those with limited positive evidence (+) with caution (Table 4). However, our recommendation of specific tools was dependent on the context in which the tools were developed and validated. For example, the well-validated measurement property in one study may not be the same in another location or cultural context. Therefore, future research should focus both on improvements of current tools and their validation in different contexts.

## Appendix 1

**Table 5:** Search strategies in PubMed

	Concept 1	Concept 2	Concept 3	Concept 4
	Key mental health disorders and mental health	3 aspects of MHL	Assessment tool	Study type
OR	“Mental Disorders”[Mesh: noexp] OR “mental health”[Mesh: noexp]	“health education”[tiab]	assessment*[tiab]	Reliability[tiab]
	“Substance-related disorders”[Mesh] OR substance use disorder*[tiab] OR “substance abuse”[tiab] OR “substance misuse”[tiab] OR “substance dependence”[tiab]	“health education”[Mesh]	evaluat*[tiab]	effective*[tiab]
OR	anxiety disorder*[tiab] OR “anxiety disorders”[Mesh] OR “generalized anxiety disorder”[tiab] OR “separation anxiety disorder”[tiab] OR “social phobia”[tiab] OR “specific phobia”[tiab] OR “panic disorder”[tiab] OR “posttraumatic stress disorder”[tiab]	“mental health literacy”[tiab]	measur*[tiab]	efficac*[tiab]
OR	disruptive behavior disorder*[tiab] OR “attention deficit and disruptive behavior disorders”[Mesh] OR “conduct disorder”[tiab] OR “oppositional defiant disorder”[tiab]	“health knowledge”[tiab]	test*[tiab]	“program evaluation”[Mesh] OR “program evaluation”[tiab]
OR	“unipolar depression”[tiab] OR “major depressive disorder”[tiab] OR depression[tiab] OR “depressive disorder”[Mesh] OR “depression”[Mesh]	“health curriculum”[tiab]	scale*[tiab]	Validity[tiab]
OR	“attention deficit hyperactivity disorder”[tiab] OR ADHD[tiab]	“mental health awareness”[tiab]	assessment tool*[tiab]	
		awareness[Mesh]	psychometrics[Mesh] OR psychometrics[tiab]	
OR		“attitude to health”[Mesh]	questionnaires[Mesh] OR questionnaire*[tiab]	
OR			survey*[tiab]	
OR		stigma[tiab]		
OR		discrimination[tiab]		
		“help seeking behavior”[tiab] OR “seeking help”[tiab]		

* means various types of suffix added at the end of a word to form a derivative, e.g., -ation, -ing, and -fy

## Appendix 2

**Table 6:** Quality criteria of measurement properties [18–20]

Property	Quality criteria	Rating
Reliability		
Internal consistency	(Sub)scale unidimensional AND Cronbach’s alpha(s) ≥ $0.70	+
	Dimensionality not known OR Cronbach’s alpha not determined	?
	(Sub)scale not unidimensional OR Cronbach’s alpha(s),0.70	-
Reliability	ICC/weighted Kappa ≥ $0.70 OR Pearson’s r ≥ 0.80	+
	Neither ICC/weighted Kappa, nor Pearson’s r determined	?
	ICC/weighted Kappa ≤ 0.70 OR Pearson’s r ≤ 0.80	-
Measurement error	MIC > SDC OR MIC outside the LOA	+
	MIC not defined	?
	MIC ≤ SDC OR MIC equals or inside LOA	-
Validity		
Content validity	The target population considers all items in the questionnaire to be relevant AND considers the questionnaire to be complete	+
	No target population involvement	?
	The target population considers items in the questionnaire to be irrelevant OR considers the questionnaire to be incomplete	-
Structural validity	Factors should explain at least 50 % of the variance	+
	Explained variance not mentioned	?
	Factors explain < 50 % of the variance	-
Hypothesis testing (construct validity)	Correlation with an instrument measuring the same construct ≥0.50 OR at least 75 % of the results are in accordance with the hypotheses AND correlation with related constructs is higher than with unrelated constructs	+
	Solely correlations determined with unrelated constructs	?
	Correlation with an instrument measuring the same construct <0.50 OR <75 % of the results are in accordance with the hypotheses OR correlation with related constructs is lower than with unrelated constructs	-
Criterion validity	Correlations with the gold standard is ≥0.70	+
	Correlations with the gold standard is unknown	?
	Correlations with the gold standard is <0.70	-
Responsiveness		
Responsiveness	(Correlation with an instrument measuring the same construct ≥0.50 OR at least 75 % of the results are in accordance with the hypotheses OR AUC ≥0.70) AND correlation with related constructs is higher than with unrelated constructs	+
	Solely correlations determined with unrelated constructs	?
	Correlation with an instrument measuring the same construct <0.50 OR <75 % of the results are in accordance with the hypotheses OR AUC <0.70 OR correlation with related constructs is lower than with unrelated constructs	-

## Appendix 3

**Table 7:** Levels of evidence for the overall quality of the measurement property [18–21]

Level	Rating	Criteria
Strong	+++ or ---	Consistent findings in multiple studies of good methodological quality OR in one study of excellent methodological quality
Moderate	++ or -	Consistent findings in multiple studies of fair methodological quality OR in one study of good methodological quality
Limited	+ or –	One study of fair methodological quality
Conflicting	+/−	Conflicting findings
Unknown	x	Studies of poor methodological quality and studies with indeterminate quality of measurement properties
